# Effect of Hot Isostatic Pressure on the Microstructure Evolution of Ti-22Al-25Nb Alloy Formed by Selective Laser Melting

**DOI:** 10.3390/ma18122806

**Published:** 2025-06-14

**Authors:** Jingjun He, Haiou Yang, Linhao Huang, Jingyu Man, Yuhan Wu, Xin Lin

**Affiliations:** 1 State Key Laboratory of Solidification Processing, Northwestern Polytechnical University, Xi’an 710072, China; 2023261216@mail.nwpu.edu.cn (J.H.); manjy1999@163.com (J.M.); wuyuhan@mail.nwpu.edu.cn (Y.W.); 2 Shenzhen Research Institute of Northwestern Polytechnical University, Northwestern Polytechnical University, Shenzhen 518057, China; 3 Xi’an Thermal Power Research Institute Co., Ltd., Xi’an 710054, China; h980103@163.com

**Keywords:** Ti-22Al-25Nb, selective laser melting (SLM), hot isostatic pressing (HIP), microstructure

## Abstract

The density of SLMed (Selective Laser Melting) Ti-22Al-25Nb alloy was improved through hot isostatic pressing (HIP) treatment, and the influence of HIP and solution aging on the microstructure of Ti-22Al-25Nb alloy in the as-deposited state was examined. The results indicate that following (1100 °C + 300 MPa)/3 h-HIP, the specimen densities have risen to 99.71%, porosity has markedly decreased, and internal flaws have been eradicated. Microstructural analysis reveals a significant presence of GBα_2_ (GB, Grain Boundary) along grain boundaries, with GBLO + α_2_ (GBL, Grain Boundary Lath; O, Orthorhombic) laths extending parallel from the grain boundaries into the intragranular region. Additionally, a limited number of cross or snowflake O + α_2_ lath clusters and acicular O phases are precipitated within the B2 (B, Body-centered cubic) phase in the HIPed state, characterized by isotropic and linear grain boundaries. The GBLα_2_ and GBLO exhibit two growth modes: sympathetic nucleation and interfacially unstable nucleation. During the solid solution treatment following HIP, as the solid solution temperature rises, the acicular O phase, GBLO, lath O phase, lath α_2,_ and GBα_2_ sequentially dissolve, increasing the volume fraction of the B2 phase. After HIP, the aging microstructure is primarily characterized by the proliferation of the acicular O phase precipitated from the B2 phase and retaining the lath O phase in a solid solution. The precipitation of GBLO in the original solid solution is suppressed, and the GBLα_2_ in the original solid solution partially decomposes into rimO, resulting in coarse grain size and significant internal decomposition of α_2_. Following solution treatment and aging at 920 °C, the proliferation of the acicular O phase enhances ductility, resulting in ideal overall characteristics with a yield strength (YS) of 760.81 MPa, ultimate tensile strength (UTS) of 869.32 MPa, and elongation (EL) of 2.683%. This study demonstrates that the HIP treatment and the modification of solution aging parameters can substantially increase the density and refine the microstructure of Ti-22Al-25Nb alloy, hence enhancing its mechanical properties.

## 1. Introduction

The Ti-22Al-25Nb alloy, distinguished by its ordered crystal structure and excellent structural stability, exhibits remarkable qualities, including elevated high operating temperature, specific strength, specific modulus, outstanding creep resistance, and oxidation resistance. These attributes render it a highly promising aeronautical material both domestically and internationally [1,2,3]. However, the inherent brittleness of Ti-22Al-25Nb alloy and the challenges associated with machining intricate multi-curved surfaces [4,5,6,7] have constrained its utilization in engineering domains [8,9,10,11]. Additionally, the progression of aircraft technology has led to a growing inclination towards massive, thin-walled, intricately linked structures, hence imposing more rigorous demands on near-net-shape forming procedures for the relevant materials [12]. Selective laser melting (SLM), a high-precision metal additive manufacturing technique, provides the benefit of attaining near-net-shape formation without the need for expensive tools or molds, in contrast to conventional manufacturing processes [13,14,15,16,17]. This technology offers a viable method for producing brittle materials and refractory alloys with intricate architectures [18,19,20,21].

In recent years, global experts have methodically investigated the process parameters of the Ti-22Al-25Nb alloy produced by SLM [20]. Qiang et al. [22] conducted a comprehensive analysis of the effect of scanning speed on the microstructure and mechanical properties of Ti-22Al-25Nb alloy. They discovered that augmenting the scanning speed enhances the cooling rate of the liquid melt, consequently diminishing the grain size of the β phase, elevating the dislocation density, and facilitating the precipitation of the O (Orthorhombic) phase along grain boundaries, thereby enhancing the alloy’s properties. Yang et al. [23] employed SLM technology to fabricate Ti-22Al-25Nb alloy and analyzed the properties of lath, acicular, and grain boundary precipitates following various heat treatments. It was found that following the solid solution treatment at 1000 °C, the maximum ultimate tensile strength of the alloy samples rose to 820 MPa. Prior research indicated that the Ti-22Al-25Nb alloy was fabricated by SLM technology, and following solution aging treatment, the resultant sample density achieved was 98.4%. The microstructural evolution and its impact on mechanical properties at different temperatures during the solid solution and unified aging treatment were systematically examined for the SLM-fabricated Ti-22Al-25Nb alloy [24]. The results indicated the viability of fabricating Ti-22Al-25Nb alloy through SLM technology and the successful application of heat treatment. Nevertheless, additional research has demonstrated that heat treatment alone cannot eradicate all defects intrinsic to the characteristics of the SLM process [25]. The mechanical properties are acutely sensitive to internal porosity and crack defects [26,27]. Consequently, enhancing the performance and longevity of SLMed Ti-22Al-25Nb components hinges on augmenting their density and regulating their microstructure.

Hot Isostatic Pressing (HIP) can eliminate internal defects inside Ti-22Al-25Nb, enhance its density, modify its microstructure, and ultimately augment its mechanical properties [27,28]. For example, Polozov et al. [29] investigated the effects of HIP, annealing, and aging across various phase zones on the deposited microstructure and phase composition of SLMed Ti-22Al-25Nb. It was determined that HIP at 1050 °C resulted in a fully dense material with a relative density of 99.9%, while the Nb (Niobium) particles remained undissolved and an α_2_(Ti_3_Al) phase developed around them. Polozov et al. [30] conducted a comparative analysis of the effects of annealing and HIP on the microstructure and properties of SLMed Ti_2_AlNb alloy. They noted that following HIP, the alloy’s tensile strength was markedly enhanced, achieving a room-temperature tensile strength of 1030 MPa. Polozov et al. [31] also employed SLM to fabricate Ti_2_AlNb-based alloy specimens with graded microstructures and conducted HIP processing to investigate the potential for eliminating microstructural inhomogeneity. The findings indicate that HIP can eradicate residual microstructural defects and markedly enhance tensile properties, achieving tensile strengths of 1027 MPa, 860 MPa and 770 MPa at room temperature, 650 °C and 700 °C, respectively.

In summary, the utilization of SLM, a near-net forming technology, effectively alleviates the intrinsic brittleness and processing difficulties of Ti-22Al-25Nb alloy. Simultaneously, HIP diminishes internal defects and improves tensile properties. Therefore, this paper conducted HIP treatment on Ti-22Al-25Nb components produced by SLM, based on prior research, and examined the effect of subsequent aging solution treatment on the microstructure of these components to facilitate effective microstructural and performance optimization.

## 2. Materials and Methods

**Figure 1:**
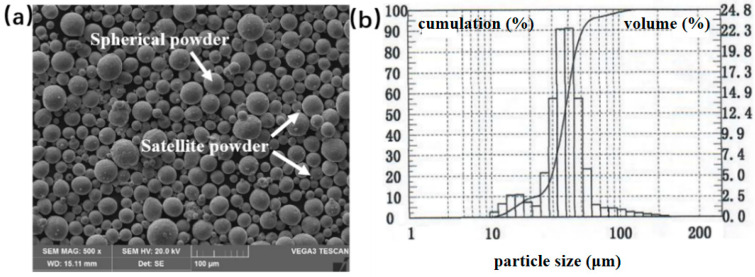
(**a**) Morphology of Ti-22Al-25Nb pre-alloyed powder; (**b**) powder particle size distribution [24].

The Ti-22Al-25Nb pre-alloyed powder was chosen for the test, and its morphology is illustrated in Figure 1a, indicating that the powder exhibits a favorable spherical shape with a minimal quantity of satellite powder adhering to its surface. The powder particle size distribution graph in Figure 1b indicates a normal distribution, predominantly concentrated in the range of 15~53 μm, with an average particle size of 38.5 μm. The powder’s chemical composition of the powder was analyzed using ICP. Table 1 indicates that the atomic percentage of aluminum is 22.27%, while that of niobium is 27.34%, satisfying the compositional criteria. The powder underwent drying in a vacuum oven at 120 °C for 2 h prior to printing.

The Ti-22Al-25Nb specimen was fabricated using the BLT-S210 apparatus (BLT, Xi’an, China). During the SLM process, high-purity argon gas was continuously supplied to maintain the ambient oxygen concentration below 500 ppm, thereby preventing material oxidation during processing. The parameters for the SLM forming process are as follows: the laser power is 175 W, the scanning speed is 800 mm/s, the powder layer thickness is 30 μm, the scanning spacing is 90 μm, the laser volume energy density is 81.02 J/mm^2^, and the scanning strategy involves rotating adjacent layers by 67°. The substrate that is formed is TC4 titanium alloy. Before formation, the surface of the substrate is subjected to sandblasting to diminish reflectivity, followed by an alcohol cleaning to eliminate oil before installation. Create multiple cubic specimens measuring 10 mm × 10 mm × 10 mm by following the aforementioned procedures.

**Figure 2:**
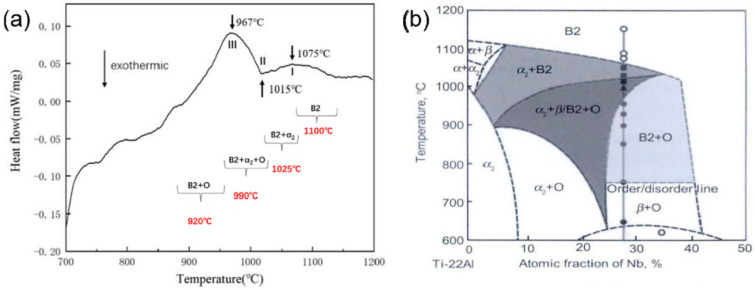
(**a**) DSC test result curve of Ti2AlNb alloy formed by SLM [24]; (**b**) Ti-22Al-xNb (at%) alloy phase diagram [32].

The HIP equipment (QIH48 URC^®^, Quintus Technologies, Shanghai, China) is utilized to investigate the effect of eliminating the porosity of the SLMed specimens and enhancing the microstructure and properties of the materials. The HIP experiment was conducted using synchronous heating and pressure elevation to a specified value, with a heating rate of 6.6 °C/min, a pressure of 300 MPa, a temperature of 1100 °C, a holding duration of 3 h, followed by furnace cooling at a rate of 10.8 °C/min. A synchronous thermal analyzer was employed to ascertain the phase transition points of the actual SLM deposited samples. Figure 2a illustrates the test curve. By integrating the DSC curve and the phase diagram of Ti-22Al-xNb alloy (Figure 2b), the precise phase transformation range and point of Ti-22Al-25Nb were established. The solid solution treatment parameters were defined by selecting solid solution temperatures of 1100 °C, 1025 °C, 990 °C, and 920 °C across the four-phase regions, with a solid solution duration of 1 h followed by water cooling. Simultaneously, considering the operational temperature range of Ti-22Al-25Nb components in practical applications, the aging treatment temperature is designated at 800 °C, with an insulation duration of 5 h and an air cooling method. The precise process parameters are delineated in Table 2.

The density of the specimens was determined using the drainage method before and after HIP. The phase composition of the specimens after different heat treatments was examined using XRD. The distribution of phase content and the characteristics of microstructural morphology of the specimens subjected to various heat treatments were examined using OM, SEM, and TEM. The nucleation and growth mechanism of the grains during HIP treatment was further examined using EBSD.

## 3. Results and Analysis

### 3.1. Effect of HIP Treatment on Microstructure of SLMed State

#### 3.1.1. Microstructure Analysis of the HIPed State

**Figure 3:**
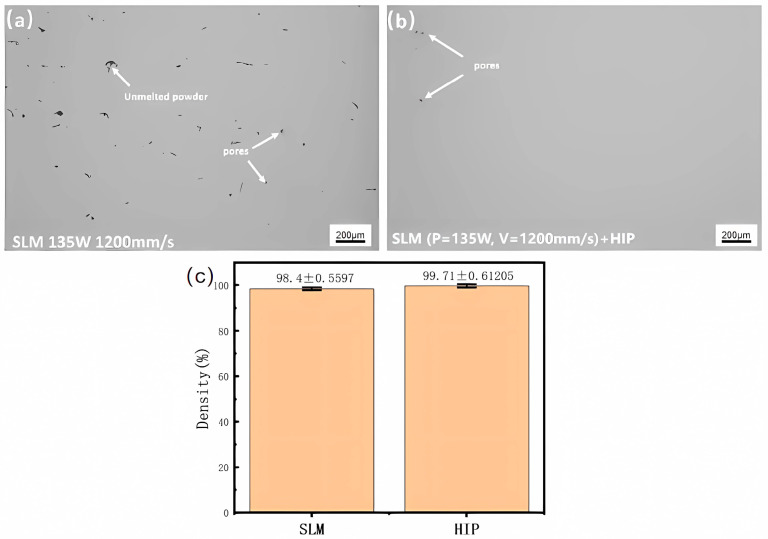
Comparison of internal porosity before and after HIP: (**a**) Density 98.40% before HIP; (**b**) Density 99.71% after HIP; (**c**) Density Error Bar Chart Before and After HIP.

Figure 3 illustrates the porosity distribution prior to and subsequent to the HIP treatment. Prior to HIP, the sample contained unmelted powders and a significant quantity of pores, exhibiting a size distribution range of 11~107 μm. In the single-phase region at 1100 °C, numerous pores in the B2 (B, Body-centered cubic) phase were eliminated under elevated temperature and pressure, resulting in the complete disappearance of large internal pores and incomplete fusion defects. Post-HIP, merely a limited quantity of pores measuring approximately 12 μm remains. The density of the SLMed Ti-22Al-25Nb specimen post-HIP was assessed using the drainage method. In comparison to the SLM state, densification rose to 99.71%, while porosity diminished by 81.88%. HIP can effectively eradicate internal porosity defects.

**Figure 4:**
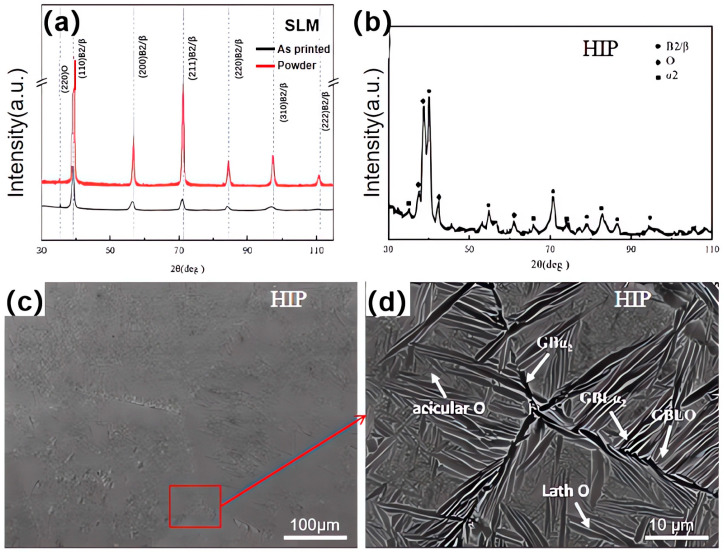
Phase microstructure analysis of the HIP state: (**a**) XRD spectrum of Ti-22Al-25Nb formed by SLM and powder; (**b**) XRD diffraction pattern of the HIP state; (**c**,**d**) BSE morphology of the HIP state.

Figure 4a presents the XRD results of the pre-alloyed powder and the SLMed Ti-22Al-25Nb alloy. Figure 4b shows the XRD results of the SLMed specimen alloy after HIP. Compared with the SLMed specimen, diffraction peaks of the O phase and the α_2_ phase emerge following HIP. During the cooling process within the B2 single-phase zone, as indicated by differential scanning calorimetry (DSC) in Figure 2a, the specimen sequentially transitions through the B2 + α_2_ two-phase zone, the B2 + α_2_ + O three-phase zone, and the B2 + O two-phase zone. Theoretically, the microstructure at room temperature consists of the B2 + O phase. However, the protracted cooling rate during furnace cooling from the B2 single-phase zone allows for the complete precipitation of the O phase and α_2_ phase. Once the α_2_ phase precipitates, it exhibits significant stability at low temperatures; therefore, the HIPed specimen comprises the B2 + O + α_2_ three phases.

The microstructure of the HIPed specimen was characterized by SEM, and the backscattered morphology of the XOY surface in the HIPed specimen is depicted in Figure 4c. In the backscattered morphology, the α_2_ phase appears black, the matrix B2 phase appears light gray, and the O phase appears dark gray as a result of the varying Nb element contents in the three phases. The grains of the SLMed specimen are small and primarily in the form of strips with obvious traces of melt channels. However, after HIP, the grains become isometric, and the grain boundaries become straight. The average grain size increased from 13.52 μm to 317.47 μm following the HIP treatment, as determined by the statistics of the grain size of multiple histograms in ImageJ (https://imagej.net/ij/). The reason for this is that the insulation in the B2 single-phase zone lacks the pinning effect of the grain boundary GBα_2_ (Grain Boundary) phase, which leads to rapid grain growth. It is evident from the enlarged image of the grain boundary junction in Figure 4d that the room temperature microstructure consists of GBα_2_ chains precipitating along the grain boundary, with a width range of 0.8 to 1.3 μm, and α_2_, O laths that are perpendicular to or at a certain angle to the grain boundary and growth in the intragranular direction. The crystal contains snowflakes or cross-lath clusters that are the result of coarse α_2_ or O phase laths. Due to the contact and collision of these adjacent laths, the size is uneven, and there are fine acicular O phases between the laths. GBLα_2_ and GBLO (Grain Boundary Lath) are the terms used to describe the α_2_ or O phase laths that are located near the grain boundary.

The primary micro characteristics of HIPed specimens are threefold: (1) the room temperature microstructure consists of a B2 + O + α_2_ three-phase structure; (2) numerous GBLO + α_2_ laths are present at the grain boundary; (3) the crystal contains multiple variants of coarser α_2_ and O laths, characterized by a greater length-to-diameter ratio.

#### 3.1.2. Comparative Analysis of Microstructure and Tensile Properties Between HIPed and Direct Solution Aging Specimens

**Figure 5:**
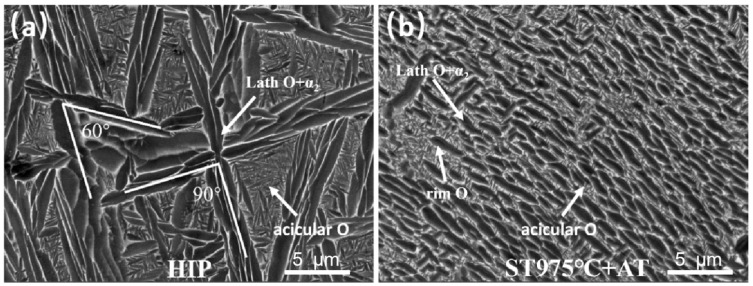
Comparison of the microstructure of Ti-22Al-25Nb alloy formed by SLM after HIP and other heat treatment: (**a**) HIP at 1100 °C; (**b**) direct solid solution aging at 975 °C.

Regarding the intracrystalline cross or snowflake clusters of coarse laths, Muraleedharan [33] categorized the intracrystalline α_2_ laths into six variants: A+, B+, C+, A− B−, and C−, with an angular relationship of approximately 60° for the variant of the same sign and about 90° for the variants of the same designation but opposite signs. Figure 5 compares the intracrystalline micro characteristics of the HIPed specimen and the solid solution aging specimen at 975 °C with similar phase compositions. It can be seen from Figure 5a that the α_2_ or O phase snowflake or cross-lath clusters of variants in the HIPed specimen adhere to this relationship, indicating the presence of each variant. Figure 5b illustrates that there are nearly no variants in the aging specimen. The initial state of the crystal is the primary factor influencing the morphological variants of the laths. As the furnace cooled during HIP, GBα_2_ preferentially precipitated. With further temperature reduction, the driving force for phase precipitation intensified, leading to the emergence of primary laths within the crystal. The interior of the crystal achieved full homogenization, reaching equilibrium after a 3-h hold in the B2 phase zone determined by DSC. The uniform energy of each precipitated phase and variant eliminates selectivity, leading to the emergence of multiple directional variants through uniform nucleation within the crystal, resulting in cross or snowflake phases. In addition, the principal lath clusters generated in the HIPed specimen, upon cooling the furnace to the B2 + α_2_ two-phase zone and B2 + α_2_ + O three-phase zone, expanded during the subsequent cooling process. The augmented driving force during cooling allowed the laths to retain a high length-to-diameter ratio.

However, the lath α_2_ and O phases in the aging state grew from the primary precipitated phases in the original solid solution specimen. There are numerous defects existing in the crystal during direct solid solution treatment in the SLM specimen, and the primary α_2_ and O phases with non-uniform nucleation at these defects adhered to the principle of minimum energy, resulting in largely uniform orientations. Furthermore, the high temperature during direct solid solution resulted in a diminished driving force for phase precipitation, and the compatibility between the α_2_ phase and B2 phase was inadequate. To reduce the interfacial energy, the primary precipitation phase was either a rod or a granule. Following aging, the encapsulation of rimO augmented the width of the primary phase while diminishing the length-to-diameter ratio. Consequently, the snowflake variant in the direct solid solution aging specimen is exclusively present in the secondary acicular O phase precipitated during aging.

**Figure 6:**
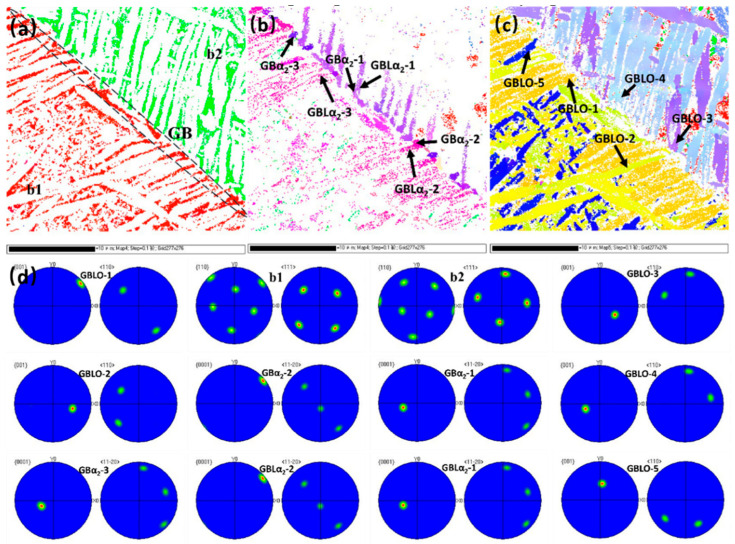
Phase distribution at grain boundaries and the GBLO + α_2_ orientation relationship: (**a**) the B2 phase distribution; (**b**) the α_2_ phase distribution; (**c**) the O phase distribution; (**d**) PF maps about the GBO, GBα_2_ and GBLO, GBLα_2_.

In order to analyze the nucleation and growth mechanism of GBL in the HIPed state, EBSD was used to characterize the orientation relationship between the GBα2 + O and GBLα2 + O at the grain boundaries. Figure 6a–c show the distribution maps of the B2, α2 and O phases within the grain boundaries, respectively. And Figure 6d shows the crystal orientation maps at specific crystal planes or orientations of the GBα2 + O and GBLα2 + O. It can be seen from Figure 6. that the grain boundary is dominated by the α2 phase, and GBLα2 + O grows from the grain boundary toward the intracrystalline direction. According to the polar diagrams, it can be seen that the B2 phase, the O phase, and the α2 phase maintain the orientation relationship of (110)B2//(001)O//(0001)α2, [111]B2//[110]O//[11−20]α2 in the HIPed state. According to this orientation relationship, it can be inferred that GBα2-1 belongs to B2 grains and GBα2-2 belongs to B1 grains, in which GBLα2-1, GBLα2-2 have the same orientation as their corresponding GBLα2-1, GBLα2-2, respectively, i.e., interfacially unstable nucleation; GBLα2-3 is oriented inconsistently with GBLα2-2 but satisfies the orientation relationship with b1 grains as shown in Figure 6a, i.e., sympathetic nucleation. Similarly, GBLO-1 and GBLO-4 satisfy (001)O//(0001)α2 and [110]O//[11−20]α2 orientation relationship with GBα2-2 and GBα2-1 respectively, which are interfacially unstable nucleation, while GBLO-2, GBLO-3, and GBLO-5 have different orientations and thus precipitate in the sympathetic nucleation. Overall, both the two growth modes of GBLα2 + O are present in the HIPed state. Based on the principle of minimum energy, GBLα2 precipitates more in the interfacially unstable nucleation while GBLO precipitates more in the sympathetic nucleation.

Based on the analysis of the above precipitation behavior, the phase precipitation process during the furnace cooling in the HIP can be deduced: In the heat preservation phase, the grains of the single B2 phase undergo rapid growth. During the furnace cooling process, due to the high distortion energy rate of the grain boundary, the α_2_ phase nucleus particle is formed initially, which makes GBα_2_ precipitate in a continuous chain or an intermittent chain. In the B2 + α_2_ and B2 + α_2_ + O phase zones determined by DSC, GBLα_2_ + O precipitates at the grain boundaries, with a large number of intragranular primary laths α_2_ + O precipitates exhibiting a cross or snowflake morphology. Upon cooling to the B2 + O phase zone, acicular O precipitates form between the laths.

The authentic stress-strain curves of specimens produced at ideal settings were acquired via tensile testing following HIP. The HIPed material exhibits a yield strength (YS) of 791 MPa at room temperature, an ultimate tensile strength (UTS) of 902 MPa, and an elongation (EL) of 4.57%. In comparison to the SLMed specimens, the HIPed specimens exhibit degraded mechanical characteristics. This degradation is ascribed to the removal of residual stresses from quick solidification and fine-grained strengthening effects in the SLMed condition, coupled with significant grain coarsening and substantial precipitation of brittle phases during HIP. However, in contrast to direct solid solution aging specimens (e.g., 990 °C solution treatment followed by aging), the HIPed material demonstrates significantly improved ductility owing to the closure of internal pores, despite a reduction in strength resulting from an increased quantity of primary laths and a diminished proportion of acicular O phase. During tensile deformation, stress concentration at lath tips intensifies as lath dimensions diminish. As shown in Figure 5, the HIPed material features a coarsened acicular O phase with blunted tips, which effectively mitigates localized stress concentration and enhances ductility.

### 3.2. Effect of Solution Temperature on Microstructure of HIPed State

#### 3.2.1. Microstructure Analysis of the HIPed Solid Solution Treatment

**Figure 7:**
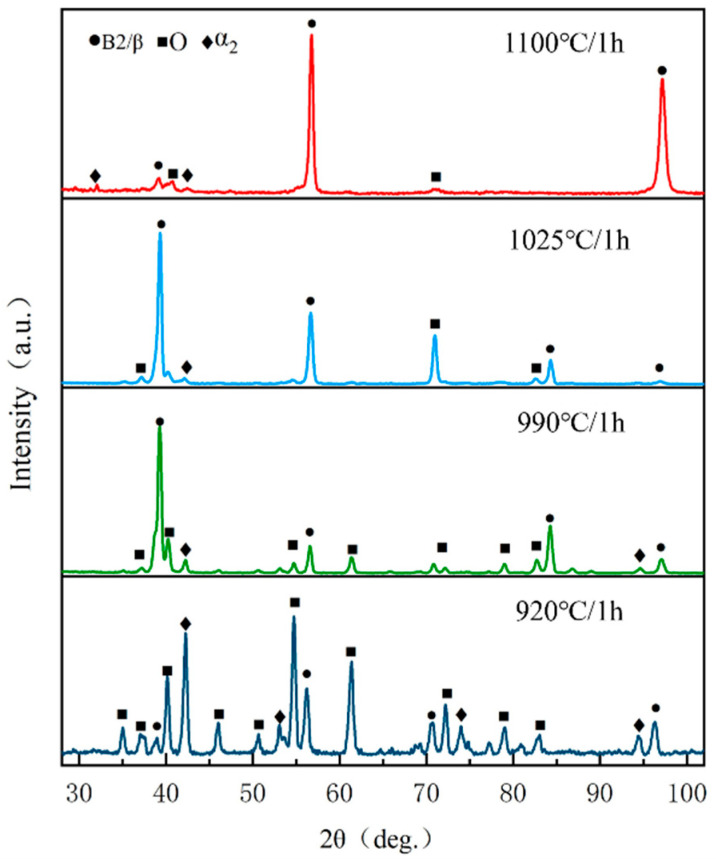
XRD diffraction patterns of the HIPed state after solid solution treatments at different temperatures.

The HIPed specimens experienced solid solution treatment at multiple temperatures within the four-phase zones defined by DSC. The solid solution temperatures were set at 1100 °C, 1025 °C, 990 °C, and 920 °C, with a duration of 1 h for the solid solution process, followed by water cooling to maintain the solid solution microstructure. Figure 7 shows the XRD data of the HIPed specimen following solid solution treatment at different temperatures. From the XRD diffraction patterns, it can be seen that all the specimens exhibit diffraction peaks corresponding to the α_2_ phase, attributable to the high stability of the α_2_ phase after the solid solution treatment. However, the encapsulation of rimO makes the α_2_ diffraction peaks low. As the solid solution temperature rises, the increased dissolution of the O phase results in a decrease in the intensity of the O phase diffraction peaks and an increase in the intensity of the B2 phase diffraction peaks.

**Figure 8:**
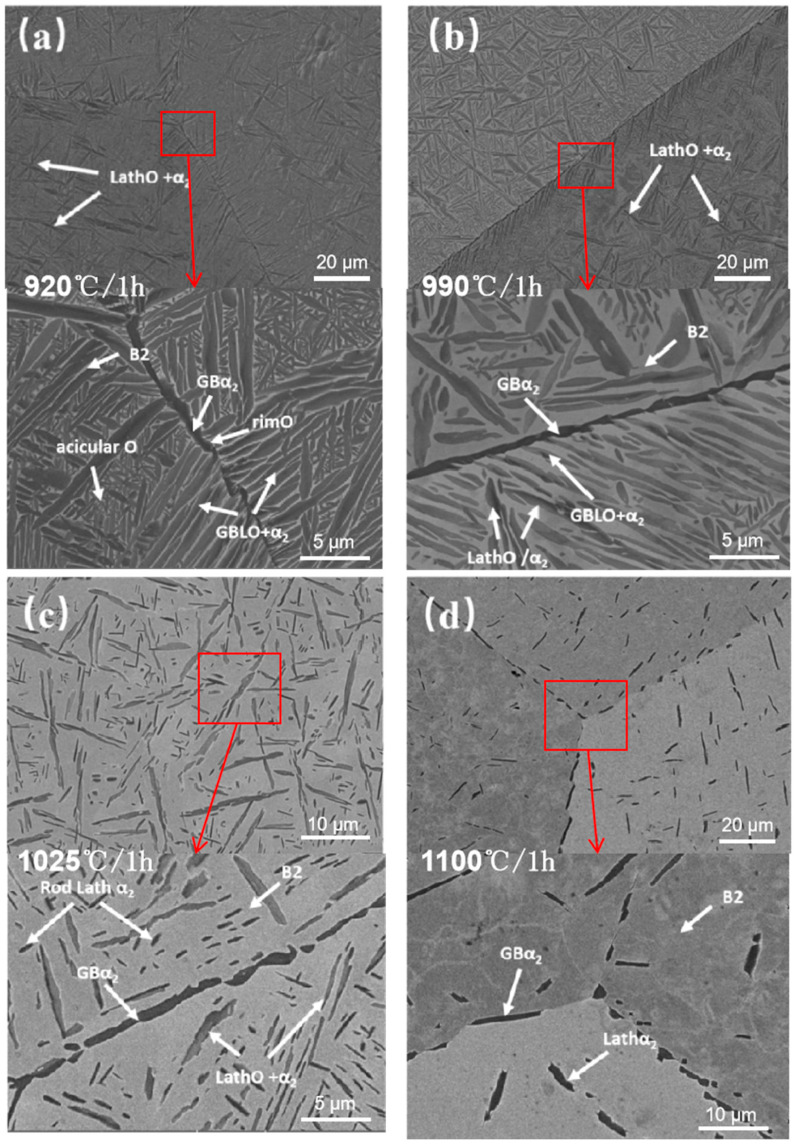
Microstructure of the HIPed state after solid solution treatments at different temperatures: (**a**) 920 °C; (**b**) 999 °C; (**c**) 1025 °C; (**d**) 1100 °C.

The BSE was employed to characterize the effect of solid solution on the microstructure of the HIPed specimen, and Figure 8 shows the microstructure of the HIPed specimen following various solid solution temperatures. Figure 8a illustrates that the microstructure consists of the black elongated α_2_ phase, the dark grey lath O phase, and the matrix B2 phase after the solid solution at 920 °C. The volume fractions of retained precipitates (VFRP) in the multiple solid solution tissues were statistically calculated by ImageJ and averaged. The VFRP in the 920 °C solid solution was approximately 70.48% (Table 3), which was not significantly different from the 71.87% seen in the HIPed specimen. The extremely fine acicular O phase interspersed among the original laths is coarsened, leading to a reduced quantity and uniform size, with a width range of 0.16 to 0.33 μm. According to the equilibrium phase diagram of Ti-22Al-xNb (at%) alloy [34], the driving force for the precipitation of the O phase increases with temperature drops during furnace cooling. However, the precipitation of the O phase necessitates elemental diffusion, and reduced temperatures impede this process; thus, lower furnace cooling temperatures result in the smaller size of the precipitated O phase. The exceptionally fine acicular O phase dissolved at a low temperature during the solid solution at 920 °C, but the residual acicular O phase coarsened throughout the solid solution processing, leading to a more uniform size. Meanwhile, the width of the intracrystalline primary lath O + α_2_ phase and the GBLO + α_2_ rose, but the length-to-diameter ratio declined. Additionally, compared to the white B2 phase and the dark gray O phase in the HIPed specimen, the color difference between the O phase and the B2 phase in the solid solution specimen decreased. Diffusion at high temperatures is more sufficient, which results in an increase in Nb content in the O phase and a reduction in the atomic ratio disparity between the O phase and the B2 phase. Theoretically, only the B2 + O phase exists at room temperature during the solid solution treatment in this phase zone [4], but the intracrystalline α_2_ phase laths and the GBα_2_ phase remain extant. The reason is that the α_2_ phase precipitated during the cooling of the HIP furnace decomposes gradually at low temperatures. The magnified observation reveals that the center of centrain laths α_2_ phase glows, and the rimO phase is wrapped around the edges, indicating that the sub-stable α_2_ phase is commencing decomposition at this temperature. However, the complete disappearance of the α_2_ phase must be in the B2 single-phase zone determined by DSC for an extended solid solution duration.

At a solid solution temperature of 990 °C, it resides inside the B2 + α2 + O three-phase zone as determined by DSC. It can be seen from Figure 8b that the number of laths decreases after the solid solution at 990 °C. Due to phase equilibrium, a large quantity of acicular O phases is dissolved, resulting in an increased content of the B2 phase and a drop in the integral number of precipitated phases to 49.22% (Table 3). The undissolved lath O phase coarsens and shortens during heat preservation, with a portion of GBLO preserved at the grain boundary. The unique α2/O phase mixed laths are observable in the high magnification microstructure. These laths differ from the α2 laths phase surrounded by rimO at 920 °C; specifically, the O phase grows on the α2 phase side, causing the formation of symbiotic laths. The solid solution treatment occurs within the B2 + α2 + O three-phase zone identified by DSC, placing the α2 phase in a metastable state, which leads to the inclusion reaction of B2 + α2→O to form the rimO phase. The enlarged image shows that GBα2 does not decompose to produce rimO, signifying that both O and α2 phases remain stable within the B2 + α2 + O three-phase zone determined by DSC. This research posits two potential explanations for the formation of symbiotic laths. Firstly, the α2→O phase transition can transpire directly within this phase zone, whereby the ordered arrangement of Ti and Nb atoms in the α2 phase, the α2 phase experiences lattice deformation, resulting in a direct transformation into the O phase. Secondly, during the solid solution, the newly created O phase nucleates and expands on the α2 phase following the dissolution of the acicular O phase. The symbiotic α2/O phase must adhere to the orientation relationship of (001) O//(0001)α2 and [110] O//[11−20]α2, irrespective of the circumstances.

Figure 8c demonstrates that a significant portion of the primary lath O + α_2_ phase in the structure is retained after exposure to 1025 °C for 1 h, with a volume fraction of the retained precipitates measuring 20.83% (Table 3), but the length-to-diameter ratio of the primary laths decreases. Theoretically, the O phase does not exist at room temperature following the solid solution treatment in the B2 + α_2_ phase zone determined by DSC; however, the enlarged view shows that the fine acicular O phase between laths has been completely dissolved, whereas the larger lath O phase remains partially intact. Concurrently, certain short rod lath α_2_ phases appear within the crystals, which are absent in the HIPed specimen. The explanation lies in the fact that during the furnace cooling of HIP at 6.6 °C/min, the B2 + α_2_ phase zone determined by DSC spans around 50 °C, leading to a brief residence time in this phase zone, which causes the incomplete precipitation of the α_2_ phase. However, during the solid solution at 1025 °C, the dissolution of the O phase and the reprecipitation of the α_2_ phase occur, i.e., the B2 + O→B2 + α_2_, resulting in the formation of short rod lath secondary precipitation phases. Following the solid solution at 1025 °C, the grain boundaries still exhibited continuous precipitation of GBα_2_, albeit with an increased breadth. This also indicates that the α_2_ phase has not been fully precipitated during furnace cooling, and the GBLO + α_2_ at a certain angle to the grain boundary, growing in the intragranular direction in the HIPed specimen, almost completely disappears after the solid solution at this temperature. From this perspective, it can be inferred that GBLO is predominant in GBLO + α_2_, aligning with the EBSD study findings presented in Figure 6.

The solid solution temperature was increased to 1100 °C, as shown in Figure 8d. The number of primary laths in the crystal significantly decreased, with only the lath α_2_ phase, acicular O phase and lath O phase completely dissolved. The GBα_2_ at the grain boundary was also dissolved, resulting in the residual GBα_2_ manifesting as an intermittent chain shape, while the volume fraction of retained precipitates post-solid solution dropped to 5.47% (Table 3). It can be seen from the figure that the size of laths in the length direction decreases significantly, the width range increases to 0.6~1.2 μm, and the length-to-diameter ratio decreases. The high surface energy of the thin laths causes the stable elements of the primary lath α_2_ phase to diffuse from regions of lesser curvature to those of greater curvature, resulting in spheroidization. Simultaneously, elevated temperatures can exacerbate the spheroidization diffusion process [35]. This spheroidization phenomenon eliminates the acicular tips of the laths, and the passivation of these tips aids in preventing the formation of tip cracks.

The above analysis indicates that the solid solution treatment in the HIPed specimen affects the structure through dissolving, whereas the solid solution temperature mainly affects the number and phase proportion of laths with different sizes in the solid solution state. With the increase of solution temperature, the VFRP increases, and the number of lath precipitates decreases.

#### 3.2.2. Effect of Solution Temperature on Tensile Properties

**Figure 9:**
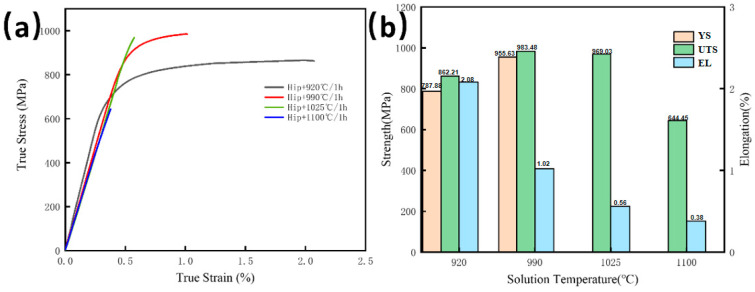
Room temperature tensile results of HIPedstate after solution treatment: (**a**) True Stress True Strain curve; (**b**) Tensile properties.

Figure 9a illustrates the real stress-strain curves at room temperature for the HIPed state following solution treatments at various temperatures, with tensile loading oriented perpendicular to the deposition direction. The tensile data depicted in the bar chart (Figure 9b) indicate that as the solution temperature increases, the UTS initially ascends and thereafter decreases, whereas elongation consistently diminishes. Specimens subjected to treatment at 920 °C and 990 °C demonstrate different yield points, accompanied by minimal strengthening during work hardening before a fracture occurs without necking. The specimen treated at 920 °C exhibits a YS of 787.88 MPa, UTS of 862.21 MPa, and EL of 2.08%. Following treatment at 990 °C, both YS and UTS rise to 955.63 MPa and 983.48 MPa, respectively, although EL decreases markedly to 1.02%. Specimens subjected to treatment at 1025 °C and 1100 °C exhibit fracture during elastic deformation, with UTS values of 969.03 MPa and 644.45 MPa and EL percentages of 0.56% and 0.38%, respectively.

The strength loss seen following 920 °C solution treatment, in comparison to the HIPed condition, is ascribed to the dissolving of ultrafine acicular O phase precipitates and the augmented volume fraction of ductile B2 phase during furnace cooling. The breakdown of the acicular O phase, which primarily contributes to strength improvement, results in mechanical deterioration. The enhancement in strength at 990 °C results from two opposing factors: (1) the decreased second-phase strengthening caused by partial dissolution of the O phase, and (2) the increased solid solution strengthening due to elevated solute saturation in the B2 matrix. The preserved GBα_2_ phases efficiently impede grain formation, whereas solute atoms and the interlath B2 phase obstruct dislocation movement, hence enhancing strength. The elevated B2 phase fraction among laths significantly diminishes ductility. Raising the solution temperature to 1025 °C enhances B2 phase supersaturation, thus augmenting strength. At 1100 °C, the pronounced supersaturation of the B2 phase significantly reduces its compatibility in coordinated deformation with the interlath α_2_ and GBα_2_ phases, leading to substantial strength degradation.

### 3.3. Effect of Aging at 800 °C on the Microstructure of the HIPed State

#### 3.3.1. Microstructure Analysis of the HIPed Solution Aging Treatment

**Figure 10:**
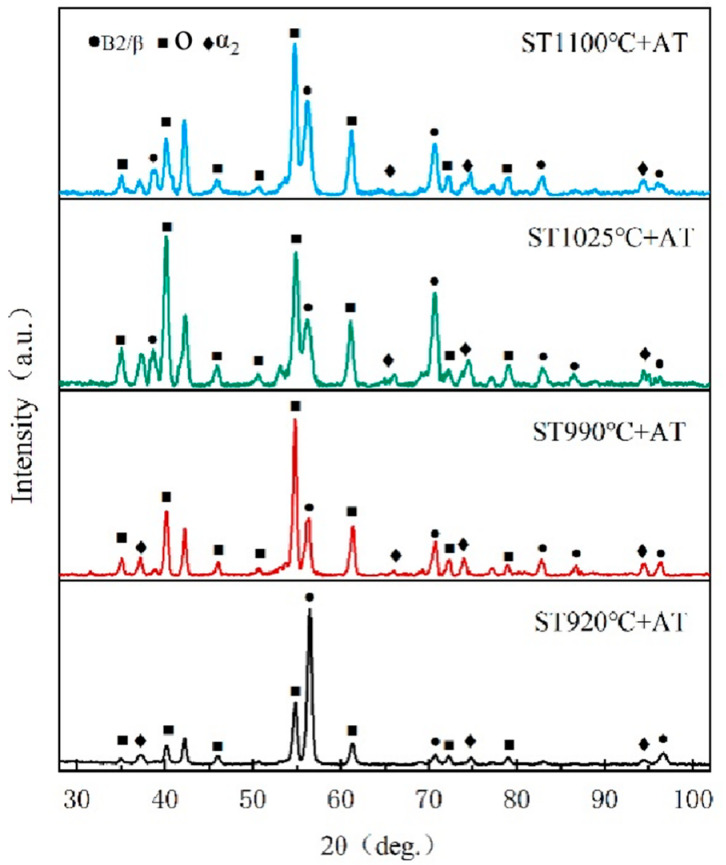
XRD diffraction pattern of the HIPed state after solution aging treatments at different temperatures.

The microstructure after solid solution treatment at different temperatures was analyzed in the previous section, revealing that the solid solution temperature mostly influences the quantity and phase proportion of primary laths. In this section, the specimens underwent aging treatment at 800 °C for 5 h after the solid solution, followed by air cooling. Figure 10 presents the XRD test results of the HIPed specimens after solution aging at different temperatures. The XRD diffraction pattern indicates that, except for the 920 °C solution aging, the O phase diffraction peaks in the other specimens intensify and dominate, which is because the aging is carried out in the O phase precipitation phase zone determined by DSC, where the B2→O transformation mainly occurs, resulting in the generation and precipitation of a large number of O phases. Due to the difficulty in decomposing the α_2_ phase at low temperatures, the various α_2_ phases retained during solid solution treatment remain detectable post-aging treatment. However, the rimO generated by the inclusion reaction during aging treatment can lead to a decrease in the α_2_ phase diffraction peak strength.

**Figure 11:**
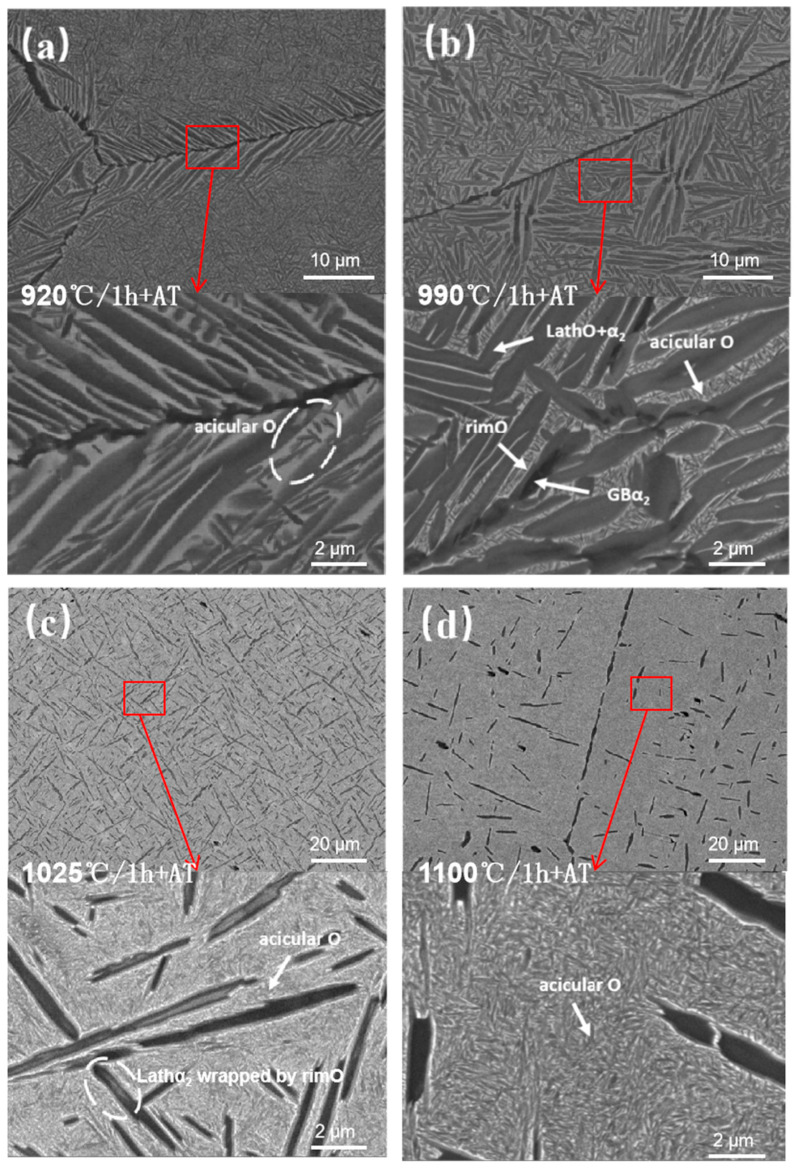
Microstructure of the HIPed state after solution aging treatments at different temperatures: (**a**) 920 °C; (**b**) 999 °C; (**c**) 1025 °C; (**d**) 1100 °C.

Figure 11 shows the SEM microstructure of the HIPed specimens after different solution aging treatments. Compared with the solid solution specimens, the microstructure of the aging state undergoes a significant increase in the volume fraction of the acicular O phase and its dispersed distribution. The B2 phase matrix exhibits enhanced brightness as a result of the elevated Nb concentration. Figure 11a illustrates the microstructure of the 920 °C solid solution state after aging. The internal microstructure is not markedly distinct from that of the solid solution state. The white circle in the figure demonstrates a smaller acicular O phase precipitated in certain regions of the matrix, with the volume fraction of the precipitated phase increasing by 2.15% after aging (Table 4). The limited spacing between acicular O phases in the solid solution state primarily facilitates the expansion and coarsening of the O phase after aging treatment while inhibiting the precipitation of GBLO in the original solid solution. The size of the acicular O phase in the aging state exhibits greater uniformity than in the solid solution state.

As shown in Figure 11b, after 990 °C solution aging, a large number of the acicular O phases are dispersed and precipitated within the interstice between the lath O phase and the α_2_ phase retained from the solid solution treatment. Their length is only half that of the fine acicular O phase in the HIPed specimen, measuring around 500 nm in length and 100 nm in width. The precipitation of several acicular O phases in the B2 matrix elevates the overall volume fraction of precipitated phases from 49.22% in the solid solution specimen to 74.63% (Table 4). The preserved lath O phase from solid solution treatment coarsens, resulting in a further reduction of the length-to-diameter ratio. The rimO phase is generated around the original symbiotic lath α_2_ phase, which is entirely enclosed.

Figure 11c illustrates that there is no significant alteration in the quantity and dimensions of the lath α_2_ phase retained in solid solution treatment following solution aging treatment at 1025 °C. The quantity and dimensions of the α_2_ phase are mainly determined by the solid solution temperature. It was discovered that the lath α_2_ phase decomposed and was encircled by the rimO phase. The precipitation of rimO made an increase in lath width and a decrease in the length-to-diameter ratio. The expanded image reveals that some α_2_ phase has decomposed into the O phase, resulting in a reduction in internal contrast. After aging, the integral number of acicular O phase precipitation is approximately 51.76%, while the total volume fraction of precipitated phases is roughly 73.78% (Table 4).

The microstructure of the aging specimen subjected to solution aging treatment at 1100 °C is depicted in Figure 11d, exhibiting a substantial quantity of distributed acicular O phase precipitates inside the B2 matrix, with a volume fraction of about 69.18% and a total precipitated phases volume fraction of around 75.46% (Table 4). This organization closely resembles the microstructure of the specimen directly subjected to solution aging at 1025 °C from the SLM specimen. The volume fraction of the α_2_ phase was unchanged before and after aging treatment, as only a small number of the primary lath α_2_ phase was retained during the solid solution treatment in the single-phase zone. Overall, as the solid solution temperature rises, VFRP remains largely constant while the number of acicular O phases increases. Subsequent to aging treatment, the homogeneous dispersion of acicular O phase precipitates was observed, while the rimO layer enveloping the α_2_ phase effectively mitigated stress concentration. This rimO phase enhances crack resistance by buffering thermal mismatch stresses, inducing crack deflection at interfaces [36], and impeding environmental diffusion-induced embrittlement [37], thereby preventing microcrack initiation and catastrophic brittle fracture [38].

Compared with the microstructure of the specimens directly kept at the same solution aging heat treatment system from the same SLM states, this experiment only shows a small number of discontinuous dissolution phenomena in the solution aging state at 1100 °C, and no blocky discontinuous dissolution is observed in the rest of the heat treatment system. In the HIPed state at 990 °C solution aging, for example, the GBα_2_ precipitated during furnace cooling was completely retained after solid solution, as shown in the white circle in Figure 11c. The phenomenon of internal decomposition of the α_2_ phase into the O phase during aging treatment is more serious, which is different from the decomposition of the α_2_ phase into the flocculent O phase during direct solution aging treatment in the SLM state. The α_2_ phase persists in a metastable state during the solid solution treatment from the HIPed state, and a considerable amount of Nb element already exists inside it. Although rimO can hinder its diffusion during the aging treatment, the internally saturated Nb element reduces the diffusion demand of distant elements to grain boundaries. At the same time, partial dissolution of GBLO + α_2_ resulted in uniform solubility of elements near GBα_2_. These factors prevent a concentration differential of Al and Nb elements near the grain boundaries, thus avoiding discontinuous dissolution phenomena. As shown in Figure 11d, discontinuous dissolution only existed in a small amount at the grain boundaries after the solution aging treatment at 1100 °C. The lath α_2_ retained after the solid solution treatment minimizes the concentration difference between the B2 phase and O phase in the matrix, and the grains have been coarsened to hundreds of microns, so the acceleration of grain borders on diffusion is minimized. These factors also, to some extent, prevent the occurrence of discontinuous dissolution caused by large temperature differences during the solution aging.

#### 3.3.2. Effect of HIPed 800 °C Aging on the Tensile Properties

**Figure 12:**
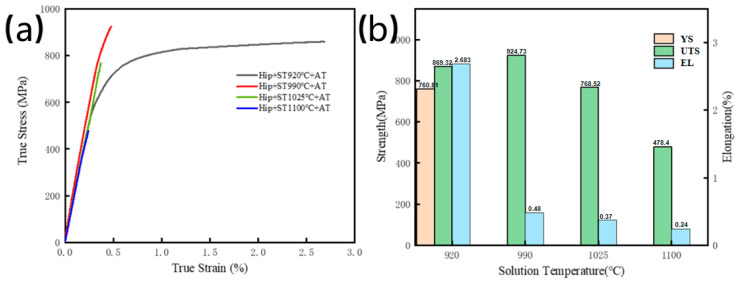
Mechanical properties of HIPed samples after solution and aging treatment: (**a**) True Stress True Strain curve; (**b**) Tensile properties.

Tensile tests at room temperature were performed on HIPed specimens that underwent solution treatment and aging at 800 °C for 5 h. Figure 12a illustrates the real stress-strain curves, indicating that only the specimen treated and aged at 920 °C displayed a definitive yield point, whilst the others failed during plastic deformation. As summarized in the bar chart (Figure 12b), the elongation decreased monotonically with increasing solution temperature, whereas UTS initially increased before subsequently declining. Compared to the solution-treated state, the specimen that experienced solution treatment and aging at 920 °C exhibited a diminished YS of 760.81 MPa but an enhanced UTS of 869.32 MPa and EL of 2.683%. Four specimens’ solution-treated at 990 °C, 1025 °C, and 1100 °C, both UTS and EL deteriorated, with respective values of 924.73 MPa, 0.48%, 768.52 MPa, 0.37% and 478.40 MPa, 0.24%.

The microstructure of the specimen treated at 920 °C and subsequently aged remained analogous to the solution-treated specimen, showing minimal precipitation of the acicular O phase. The slight coarsening of the O phase and improved uniformity of lath dimensions during aging reduced the interfacial density between laths and the B2 matrix, thereby increasing the average free slip distance for dislocations and improving ductility. In contrast, the specimen treated at 990 °C displayed significant acicular O phase precipitation in interlath areas, creating numerous B2/O contacts that obstructed dislocation movement, leading to maximum strength but considerable loss of ductility. At 1025 °C, solution treatment within the α_2_ phase stability region caused coarsening of GBα_2_. The coarse GBα_2_ phases, retained after aging, cracked readily during tensile deformation. Concurrently, high acicular O phase precipitation reduced matrix ductility, disconnecting the GBα_2_ from the matrix and drastically lowering strength. The specimen treated at 1100 °C showed further increases in the volume fraction of the acicular O phase. The inadequate crack propagation resistance of this O phase-dominated microstructure led to unimpeded crack growth and degradation of both strength and ductility.

In summary, the HIPed specimen, which had a solution treatment at 920 °C followed by aging at 800 °C for 5 h, exhibited enhanced room temperature ductility compared to the directly solution-treated and aged as-deposited condition, attributable to the coarser acicular O phase and near-complete densification. At different temperatures, despite the elimination of internal flaws, coarse grains, and detrimental precipitates, a significant decline in mechanical characteristics was observed.

## 4. Summary and Conclusions

1. Following (1100 °C + 300 MPa)/3 h HIP treatment, the unfused flaws inside the specimen in the SLM state were eliminated, and the pores were sealed. The HIPed state segregated the GBα_2_ at the grain borders, with the GBLO + α_2_ laths developing parallel from the grain boundaries toward the intragranular region, alongside the cross or snowflake O + α_2_ lath clusters and acicular O phase within the B2 phase. The GBLO + α_2_ was precipitated through interfacially unstable nucleation and sympathetic nucleation, according to a particular orientation relationship.
2. The volume fraction of the B2 phase in the HIPed state increases with rising solid solution temperature, sequentially dissolving the acicular O phase, GBLO, lath O phase, lath α_2_, and GBα_2_ in order. The dimensions of the acicular O phase exhibit greater uniformity following solid solution at 920 °C, α_2_/O symbiotic laths emerge after solid solution at 990 °C, the reprecipitation of bar α_2_ transpires during solution treatment at 1025 °C, and the GBα_2_ dissolves in an intermittent chain subsequent to solid solution at 1100 °C.
3. Following HIP, the aging microstructure is primarily characterized by the proliferation of the acicular O phase precipitated from the B2 phase and the retained lath O phase inside a solid solution. The precipitation of GBLO in the original solid solution is suppressed, and the GBLα_2_ in the original solid solution partially decomposes into rimO, resulting in coarse grain size and significant internal decomposition of α_2_. The phenomenon of discontinuous dissolution, attributed to a continuous distribution of the GBα_2,_ disappears. However, a minor degree of discontinuous dissolution persists due to the significant temperature differential during the solution aging at 110 °C.
4. Compared to direct solution aging specimens, the HIPed condition demonstrates increased densification but a coarser-grained structure, resulting in diminished strength and a notable boost in ductility. Following solution treatment and aging at 920 °C, the proliferation of the acicular O phase enhances ductility, resulting in optimum overall characteristics with YS of 760.81 MPa, UTS of 869.32 MPa, and EL of 2.683%.

## Figures and Tables

**Table 1:** Chemical composition of Ti-22Al-25Nb pre-alloyed powder (at.%) [24].

Element	Al	Nb	Ti
at.%	22.27	27.34	50.39

**Table 2:** Heat treatment parameters after HIP.

Specimens	Solution	Cooling Mode	Aging	Cooling Mode
ST920	920 °C/1 h	water-cooling	—	—
ST990	990 °C/1 h	water-cooling	—	—
ST1025	1025 °C/1 h	water-cooling	—	—
ST1100	1100 °C/1 h	water-cooling	—	—
ST920 + AT	920 °C/1 h	water-cooling	800 °C/5 h	air-cooling
ST990 + AT	990 °C/1 h	water-cooling	800 °C/5 h	air-cooling
ST1025 + AT	1025 °C1 h	water-cooling	800 °C/5 h	air-cooling
ST1100 + AT	1100 °C/1 h	water-cooling	800 °C/5 h	air-cooling

**Table 3:** Volume fraction and main characteristics of precipitated phases after different solid solution treatments.

Specimens	Solution	VFRP (%)	Main Characteristics of Precipitates
ST920	920 °C/1 h	70.48	α_2_ + O + B2, acicular O dissolved
ST990	990 °C/1 h	49.22	α_2_/O phase mixed laths, B2 increased
ST1025	1025 °C/1 h	20.83	GBLO, acicular O completely dissolved
ST1100	1100 °C/1 h	5.47	Only Lathα_2_, GBα_2_ dissolved

**Table 4:** Volume fraction and main characteristics of precipitated phases after different solid solution aging treatments.

Specimens	Solution	VFRP (%)	Main Characteristics of Precipitates
ST920 + AT	920 °C/1 h	72.63	smaller acicular O, O growth and coarsening
ST990 + AT	990 °C/1 h	74.63	abundant acicular O, Lath O coarsening
ST1025 + AT	1025 °C/1 h	73.78	Lath α_2_ wrapped rimO, α_2_ decomposed into O
ST1100 + AT	1100 °C/1 h	75.46	abundant dispersed acicular O

## Data Availability

The original contributions presented in this study are included in this article. Further inquiries can be directed to the corresponding authors.

## Abbreviations

The following abbreviations are used in this manuscript:

SLM	Selective Laser Melting
HIP	Hot isostatic pressing
GB	Grain Boundary
GBL	Grain Boundary Lath
DSC	Differential scanning calorimetry
O	Orthorhombic
B2	Body-centered cubic
VFRP	volume fraction of retained precipitates
YS	Yield Strength
UTS	Ultimate Tensile Strength
EL	Elongation
